# From basic biology to engineered therapies: the keratinocyte stem cell playbook

**DOI:** 10.3389/fmedt.2026.1763067

**Published:** 2026-02-06

**Authors:** Adnan Uddin, Mohamad Rahmani, Abdulrahim Sajini

**Affiliations:** 1 Department of Biological Sciences, Khalifa University of Science and Technology, Abu Dhabi, United Arab Emirates; 2 Department of Biology, Chemistry and Environmental Sciences, College of Arts and Sciences, American University of Sharjah, Sharjah, United Arab Emirates; 3 Bioinformatics and Computational Biology Research Group, American University of Sharjah, Sharjah, United Arab Emirates

**Keywords:** exosome, keratinocyte, regenerative medicine, skin graft, wound healing

## Abstract

Keratinocyte stem cells (KSCs) are the principal drivers of epidermal renewal, barrier maintenance, and wound repair. Their ability to alternate between self-renewal and differentiation is orchestrated by tightly integrated extrinsic and intrinsic programs that ensure tissue stability while enabling rapid regeneration after injury. This review synthesizes current understanding of KSC homeostasis through a unified framework of three interdependent “fate locks”—the identity switch (ΔNp63 ↔ Notch/IRF6-KLF4/GRHL3/OVOL), the cell-cycle lock (E2F/MYC ↔ p21/p27-RB), and the mechanotransduction lock (YAP/TAZ ↔ Hippo/LATS). We summarize how niche-derived cues—integrins/ECM, EGFR, Wnt, Notch, Ca^2^^+^/CaSR, and TGF-*β*—interface with intrinsic timers such as asymmetric division, DNMT1-UHRF1-mediated epigenetic memory, the DNA-damage response, proteostasis/autophagy, and redox signaling to steer keratinocyte fate. Building on this biological foundation, we categorize current methods for isolation and xeno-free expansion of primary human keratinocytes, emphasizing advances in defined media, feeder-free substrates, and biomimetic culture surfaces. We further review 3D and organotypic models, hydrogel-based delivery systems, and the growing portfolio of keratinocyte-derived clinical products used in wound healing. Finally, we highlight emerging applications extending beyond cutaneous repair—including immunomodulation, pigment restoration, ocular and mucosal regeneration, and acellular exosome-based therapeutics.

## Introduction

Disruptions of wound healing phases of the skin lead to chronic wound formation, excessive wound healing, substantial consumption of resources and long-term medical management (1). Extensive skin injuries—whether from burns, diabetic ulcers, or obesity-related complications—significantly impair the skin's innate regenerative capacity and pose serious risks to patient well-being (2). Non-fatal burn injuries are one of the leading causes of morbidity in the world.

According to the Global Burden of Disease 2019 estimates, burn injuries caused an estimated 8.4–9.0 million new burn cases globally, with >110,000 attributable deaths and millions of disability-adjusted life years lost, while the American Diabetes Association reports that 25% of individuals with diabetes will experience a hard-to-heal wound in their lifetime, often culminating in infection or even amputation (3, 4). Chronic wounds thus represent a growing global burden, characterized by limited treatment options and escalating costs (5).

In response, stem cell–based strategies have gained prominence for their regenerative promise, with epidermal KSCs emerging as especially attractive candidates due to their abundance, accessibility, and pivotal role in skin homeostasis and repair. Keratinocytes account for over 90% of epidermal cells, residing in the basal layer where they proliferate, then migrate outward and differentiate through the epidermis layers to re-establish barrier function and secrete cytokines that orchestrate inflammation and matrix remodeling (7). Their well-characterized markers (basal keratins K5/K14, high expression of integrins such as integrin *β*1 and integrin, transcription factors including ΔNp63*α*, and low expression of differentiation-associated markers such as K10 and involucrin) and robust *in vitro* expansion protocols further simplify their isolation and scale-up compared with other cutaneous progenitors. indeed, the vast majority of advanced wound-healing products—such as Epicel® (autologous confluent sheets), Apligraf® and OrCel® (bilayer allogeneic composites), ReCell® (autologous cell suspension spray), Dermagraft® (fibroblast-loaded scaffold), and StrataGraft® (keratinocyte/fibroblast bilayer)—leverage keratinocyte biology to enhance re-epithelialization and tissue regeneration (6). Beyond these established modalities, keratinocytes are now being investigated for an expanding array of emerging applications, including mucosal regeneration, ocular surface repair, gene-based therapies, and targeted immunomodulation.

In this review, we first outline the biological and molecular foundations of keratinocyte stem-cell homeostasis, emphasizing the extrinsic and intrinsic pathways that govern the balance between renewal and differentiation. These mechanisms are integrated into a unified “three-lock” framework encompassing identity, cell-cycle, and mechanotransduction control. Building on this foundation, we categorize current protocols for the isolation and xeno-free expansion of primary human keratinocytes, review commercial keratinocyte-based products used in wound repair, and assess recent progress in 3D and organotypic culture systems. Finally, we highlight emerging therapeutic applications—from mucosal and ocular regeneration to immunomodulation and acellular, exosome-based approaches—and discuss how these advances position keratinocytes as a central platform for next-generation regenerative medicine.

### Stem cell biology

Human skin is organized into three principal layers—the epidermis, dermis, and hypodermis—connected by the dermo-epidermal junction, a specialized multilayered basement membrane that anchors the epidermis to the underlying dermis and regulates molecular exchange. The epidermis is an avascular, continuously renewing tissue composed of two main compartments: the interfollicular epidermis (IFE) and the pilosebaceous unit, which includes hair follicles and sebaceous glands. Epidermal stem cells represent a heterogeneous population of long-lived cells that sustain epidermal homeostasis and regeneration; within this broader group, KSCs specifically denote interfollicular epidermal stem cells committed to the keratinocyte lineage. The IFE is stratified into four sequential layers—basal, spinous, granular, and cornified—each corresponding to a defined stage of keratinocyte differentiation. KSCs reside in the basal layer immediately above the basement membrane, where they proliferate and initiate the upward migration that underpins continuous epidermal turnover. Derived from the embryonic ectoderm, keratinocytes constitute approximately 90% of epidermal cells and execute a tightly regulated program of proliferation, differentiation, and barrier formation (7).

KSCs of the skin reside in specialized microenvironments—niches—comprised of multiple cell types and key regulatory cues, including extracellular matrix (ECM) components, direct cell–cell interactions, and soluble growth factors. Homeostasis of the IFE depends on KSCs to continuously replace shed cells and to mount repair responses following injury. These SCs adhere directly to the basement membrane in the lower basal layer, where they divide by a process known as population asymmetry—typically yielding one SC and one transit-amplifying (TA) progenitor cell, although symmetric divisions (producing two SCs or two TA cells) can occur under specific demands (8). TA cells proliferate for a limited number of cycles before committing to terminal differentiation and migrating upward through the spinous, granular, and cornified layers to become corneocytes. This withdrawal from the cell cycle and progression through differentiation is collectively termed keratinization. Within the basal layer, a dynamic coexistence of slow-cycling SCs and more rapidly dividing progenitor cells maintains both long-term tissue renewal and everyday turnover (9). The Keratinocyte SCs and their differentiated progeny that are migrating upwards are organized into cone-like columns recognized as epidermal proliferation units (EPU)- reflecting the organized, hierarchical nature of epidermal maintenance and migration (10). Collectively, epidermal SCs exhibit the two hallmark properties of all stem cells: self-renewal—by division within the basal niche to preserve the stem cell pool—and differentiation—upon exit from the niche, initiating the terminal program that culminates in corneocyte formation.

### Homeostasis and fate decisions

**Figure 1:**
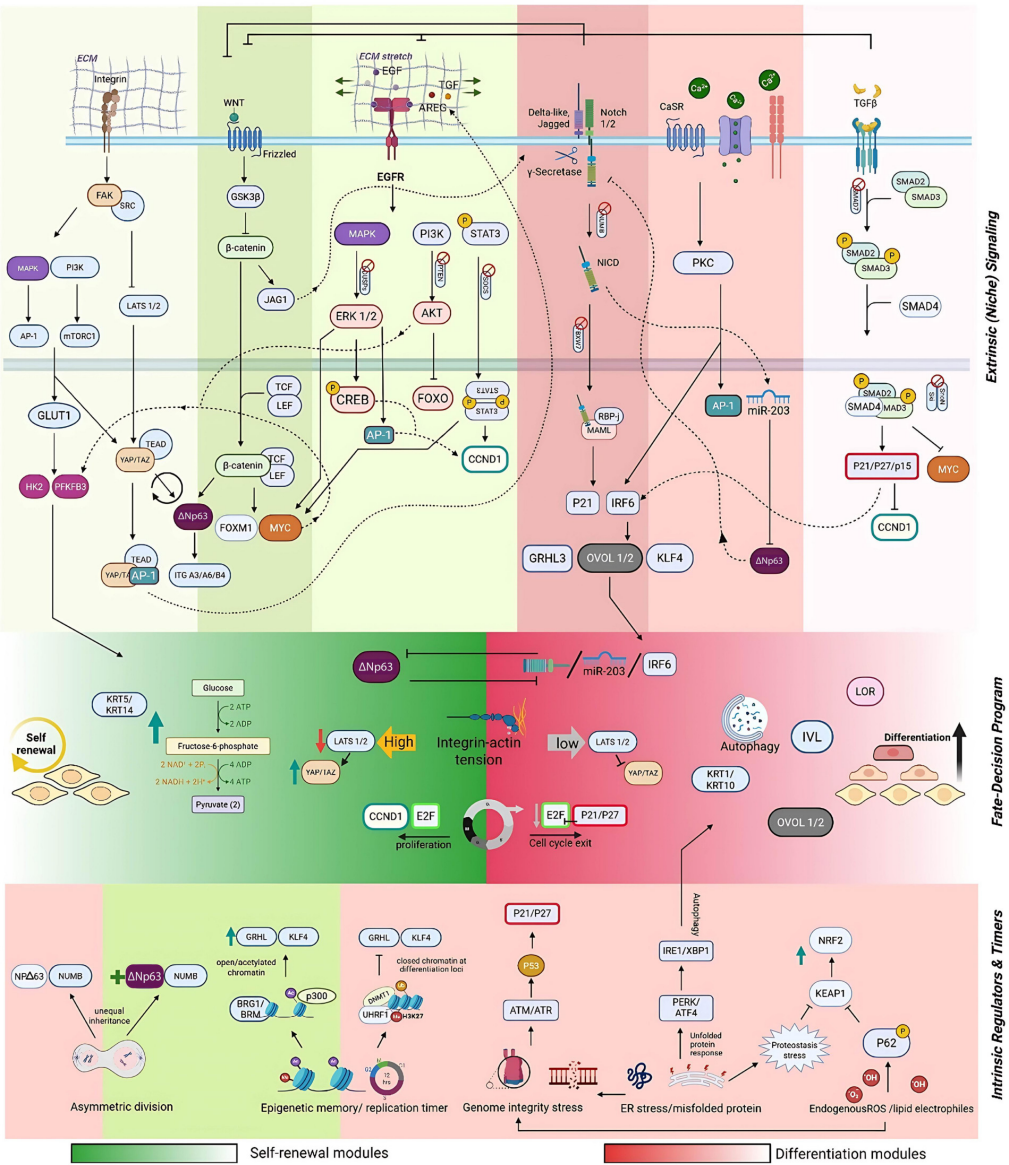
Integrated regulatory framework controlling keratinocyte stem-cell fate and homeostasis. Keratinocyte renewal and commitment arise from the integration of extrinsic niche cues (Integrin/FAK–Src, EGFR, Wnt, Notch, Ca^2+^/CaSR, TGF-*β*) and intrinsic programs (asymmetric division, DNMT1–UHRF1 epigenetic maintenance, DNA-damage checkpoints, proteostasis/autophagy, and KEAP1–NRF2 redox control). These signals converge on three interdependent “fate locks”: an identity switch (ΔNp63↔Notch/IRF6–KLF4/GRHL3/OVOL), a cell-cycle lock (E2F/MYC↔p21/p27–RB), and a mechanotransduction lock (YAP/TAZ↔Hippo/LATS). Crosstalk and feedback brakes (MIG6, SMAD7, NUMB/FBXW7) stabilize this landscape, keeping basal keratinocytes poised for renewal yet primed for rapid differentiation when cues align.

Basal keratinocytes continuously choose between self-renewal (to sustain the proliferative basal compartment) and differentiation (to construct and repair the epidermal barrier). Extrinsic niche cues—integrins/ECM, EGFR ligands, Wnt, Notch, Ca^2+^/CaSR, TGF-*β*—bias this decision, while intrinsic programs— epigenetic memory, proteostasis/autophagy, DNA-damage checkpoints, Redox/KEAP1–NRF2 switch, asymmetric division—set thresholds and timing. Mechanistically, fate resolves through a small set of convergent “locks”: an identity switch (ΔNp63 vs. Notch/IRF6-KLF4/GRHL3/OVOL), a cell-cycle gate (E2F drive vs. p21/p27-RB arrest), and a mechanotransduction gate (nuclear YAP/TAZ under adhesion/tension vs. Hippo/LATS-mediated shutdown). In what follows, each pathway is discussed in terms of where it lands on these locks to push keratinocytes toward renewal or commitment (Figure 1).

#### 1. Extrinsic (niche) signalling → fate outcome

##### Integrin/FAK–Src mechanotransduction (renewal-biased)

Engagement of basement-membrane laminins by *α*6*β*4 and *α*3*β*1 integrins triggers FAK/Src and Rho–ROCK, sustaining actomyosin tension and promoting nuclear YAP/TAZ, which cooperate with TEAD (and frequently AP-1) to amplify basal enhancers that maintain ΔNp63-centric identity, CCND1/MYC→E2F cycling, and glycolytic/mTORC1 metabolism (11). In mouse epidermis and primary keratinocytes, integrin-dependent tension is a principal determinant of YAP/TAZ nuclear localisation and clonogenicity, whereas loss of adhesion or pharmacologic FAK inhibition activates Hippo LATS1/2, expels YAP/TAZ from the nucleus, and biases cells toward commitment (12). Thus integrin signalling feeds the mechanotransduction lock and keeps cells on the renewal side until delamination reduces tension (13).

##### EGFR (ERK/AKT/STAT3) with ADAM17-dependent ligand shedding (renewal-biased; dose-dependent)

Basal keratinocytes produce membrane-tethered amphiregulin and TGF-α that are shed by ADAM17, creating potent auto/paracrine EGFR activation; downstream ERK→AP-1, PI3K–AKT→mTORC1, and STAT3 sustain proliferation, survival, and anabolic flux (14). *In vivo*, epidermal ADAM17 loss compromises barrier formation because physiological EGFR tone is required during tissue maintenance, yet reducing EGFR signalling in organotypic epidermis dampens proliferation and elevates FLG/IVL/DSG1, illustrating that the same pathway can favour differentiation when its output falls below a renewal-supporting threshold. Via AP-1, EGFR cooperates with YAP/TAZ at basal enhancers, while ligand availability and receptor down-modulation (e.g., by MIG6/CBL; see “brakes”) tune dose and duration (15).

##### Wnt/*β*-catenin (context-dependent; generally renewal-supporting)

Canonical Wnt signaling activates Frizzled and LRP5/6 co-receptors, leading to inhibition of the GSK3*β*–Axin–APC destruction complex. This stabilization of cytoplasmic *β*-catenin allows its nuclear accumulation, where it partners with TCF/LEF transcription factors to activate target genes such as MYC and CCND1, thereby amplifying progenitor proliferation and fueling E2F-driven cell-cycle progression (16). In the epidermis, *β*-catenin plays a dual context-dependent role: it is indispensable for hair follicle morphogenesis and stem-cell activation, yet in the interfollicular epidermis it primarily sustains proliferation within basal keratinocytes while being constrained by antagonistic inputs from Notch and other differentiation-promoting cues. Notably, *β*-catenin can directly induce JAG1 expression, functionally coupling Wnt-active domains to adjacent Notch-responsive territories and thus mediating lineage allocation within appendageal niches. As illustrated in Figure 1, Wnt signaling principally converges on the cell-cycle lock, reinforcing E2F and MYC activity to promote renewal, while secondarily supporting basal identity programs (ΔNp63 maintenance and YAP/TAZ nuclear localization). The strength, duration, and outcome of Wnt activity are finely tuned by the local niche architecture and cross-regulatory feedbacks with EGFR, Notch, and integrin–mechanotransduction pathways (17).

##### Notch (NICD/RBP-J/CSL/MAML) (differentiation-biased)

Engagement of membrane-bound ligands (Delta-like or Jagged) on neighboring cells activates Notch receptors through *γ*-secretase-mediated cleavage, releasing the Notch intracellular domain (NICD). The NICD then translocates to the nucleus, where it associates with RBP-J/CSL and the co-activator MAML to drive transcription of a keratinocyte commitment program. In primary human keratinocytes, p21 (CDKN1A) is a direct Notch1/RBP-J target, linking Notch activation to cell-cycle arrest. Notch signaling also induces IRF6, which functions in concert with KLF4, GRHL3, and OVOL transcription factors to establish the early differentiated gene network, and promotes miR-203, a post-transcriptional repressor of ΔNp63 (18, 19). Collectively, these outputs extinguish the basal identity program (ΔNp63^OFF^), reinforce RB hypophosphorylation and E2F inactivation, and elevate differentiation-associated transcription factors, thereby propelling keratinocytes across the commitment threshold. Additionally, cooperation with the calcineurin/NFAT pathway further stabilizes the post-mitotic state and consolidates differentiation commitment (20).

##### Calcium/CaSR→PLC*β*→PKC/AP-1 (differentiation-biased)

The suprabasal calcium gradient engages the calcium-sensing receptor (CaSR), which couples to Gq/PLC*β* signaling to trigger inositol-1,4,5-trisphosphate (IP₃)–mediated endoplasmic-reticulum Ca^2+^ release and subsequent store-operated calcium entry (SOCE) via STIM, Orai, and TRPC channels. Concurrently, diacylglycerol (DAG) activates protein kinase C (PKC), leading to phosphorylation and activation of the AP-1 transcriptional complex (21). Functional studies demonstrate that keratinocyte-specific deletion of CaSR delays epidermal barrier formation, enhances basal proliferation, and disrupts lamellar-body secretion, while pharmacologic inhibition of CaSR suppresses Ca^2+^-induced differentiation *in vitro* (22). CaSR/PKC/AP-1 also promotes miR-203, thereby helping silence ΔNp63 and synchronising identity and cell-cycle exit with the onset of KRT1/KRT10 and other spinous markers; Ca^2+^ channel composition modulates signal strength across layers (23).

##### TGF-*β*/SMAD2/3/4 (differentiation-biased)

Ligand engagement of the type I and type II TGF-*β* receptors activates SMAD2 and SMAD3, which form a complex with SMAD4 to initiate the canonical cytostatic transcriptional program. This cascade induces CDK inhibitors (p15^INK4b, p21^CIP1, p27^KIP1) and represses MYC, resulting in RB hypophosphorylation, E2F inhibition, and cell-cycle arrest prior to spinous layer entry. Negative feedback is provided by SMAD7, which attenuates signaling at both the receptor and SMAD levels (24). In keratinocytes, the TGF-*β*/SMAD axis cooperates with Notch and Ca^2+^/CaSR signaling to consolidate differentiation commitment, while MYC repression counteracts proliferative programs driven by EGFR and Wnt pathway (25). The dominant landing point is therefore the cell-cycle lock, with indirect reinforcement of identity flipping (26).

##### Crosstalk and brakes (stability of the decision)

The stability of keratinocyte fate decisions emerges from a network of mutual antagonisms and built-in pathway “brakes” that prevent erratic switching. Notch signalling restrains Wnt/*β*-catenin activity through both membrane-proximal and nuclear mechanisms, generating spatially distinct Notch-ON/Wnt-OFF territories within stratified epithelia. Conversely, *β*-catenin can induce JAG1, allowing Wnt-active cells to relay signalling to neighboring Notch-responsive cells during follicular lineage specification (16). Notch and ΔNp63 form a reciprocal toggle: Notch (and Ca^2+^/PKC) upregulate miR-203, which represses ΔNp63, whereas the basal ΔNp63 program dampens Notch responsiveness and sustains the progenitor state. To prevent overshoot and ensure homeostatic balance, pathway-specific inhibitory circuits act as molecular brakes—MIG6/ERRFI1 and CBL attenuate EGFR activity; SMAD7 provides negative feedback on TGF-*β* signalling; and NUMB/FBXW7 promote turnover of Notch receptors and NICD, limiting sustained activation (27, 28). These interlocking feedbacks and antagonisms maintain basal keratinocytes in a poised, metastable configuration just short of commitment, yet enable rapid and coordinated transitions toward differentiation when the appropriate cues converge.

#### 2. Intrinsic Regulators and Timers of Commitment (Cell-Autonomous View)

##### Asymmetric division & NUMB partitioning (intrinsic polarity as a fate splitter)

Basal keratinocytes can divide asymmetrically, producing one basal daughter that retains renewal capacity and a sister that is primed for commitment (29). The initiator is the cell's intrinsic polarity/spindle-orientation machinery—LGN (Gpsm2)–NuMA and associated complexes—that orients the mitotic spindle relative to the basement membrane. Genetic perturbation of this machinery reduces perpendicular/asymmetric divisions, impairs stratification, and shows that Asymmetric-cell-division promotes Notch-dependent differentiation in the suprabasal daughter, whereas the basal daughter inherits factors that maintain renewal (30). NUMB, an endocytic adaptor inherited unequally, antagonizes Notch in the renewing daughter, providing a second, autonomous bias within the same mitosis (30). Recent *in vivo* work confirms that modulators of spindle orientation (e.g., AGS3/Gpsm1) tune the balance of planar vs. perpendicular divisions during stratification (31).

##### Epigenetic memory/replication timer (DNMT1–UHRF1-coupled maintenance)

A second intrinsic layer is replication-coupled epigenetic maintenance. During S-phase, UHRF1 recognizes hemi-methylated CpGs and histone marks and recruits/activates DNMT1 at replication forks to restore methylation on the nascent strand (32). In epidermis, DNMT1 is enriched in basal progenitors and required for self-renewal; its depletion precipitates premature differentiation in human and mouse models. Conceptually, the DNMT1–UHRF1 system acts as a timer: as progenitors mature or DNMT1/UHRF1 levels decline, differentiation loci (including EDC genes and TFs such as KLF4/GRHL) become permissive to activation (33). Mechanistically, this maintenance arm cooperates with other repressors (e.g., PRC2/EZH2; HDAC complexes) but is not a single holocomplex; rather, parallel repressive tracks converge on chromatin compaction. Once the maintenance threshold drops, chromatin opening by p300 and BAF/SWI–SNF allows the differentiation program (34).

##### Genome-integrity stress (DDR → p53 → p21; a tunable intrinsic brake)

Keratinocytes experience endogenous genomic stress from replication overload (E2F/MYC drive), UV photolesions, and oxidative damage. This intrinsically activates the DNA-damage response (DDR): ATR at stalled forks and ATM at double-strand breaks, with downstream CHK1/CHK2 signaling, *γ*H2AX deposition, and stabilization of p53 (35). A defining epidermal output is p21^Cip1 induction, which inhibits CDKs, imposes RB-dependent E2F shutdown, and biases toward cell-cycle exit and commitment. Human keratinocyte studies show p21 is required for the squamous differentiation response to replication stress; conversely, selective contexts reveal complex p21 roles, but the dominant DDR outcome in primary keratinocytes is cytostatic and pro-commitment (36). Additional p53 targets (14-3-3*σ*/SFN, GADD45A) enforce the checkpoint. The effect is dose–time dependent: transient DDR allows repair and return to renewal; persistent DDR shifts fate toward differentiation, and severe damage triggers senescence or apoptosis.

##### Proteostasis/ER stress → UPR/ISR and selective translation (autophagy coupling)

As keratinocytes gear up for barrier production, protein-folding and secretory load increase, generating proteostasis stress—often manifest as ER stress. This activates a mild unfolded protein response (UPR) and the integrated stress response (ISR), particularly PERK–eIF2*α* phosphorylation, which globally dampens translation yet selectively maintains translation of differentiation-linked mRNAs (e.g., involucrin) via upstream ORFs or RNA features. In human keratinocytes, eIF2*α* phosphorylation is required for normal differentiation; polysome profiling shows key differentiation transcripts remain on heavy polysomes despite lower global protein synthesis. In parallel, autophagy/mitophagy is up-scaled to remodel organelles and supply substrates for envelope and lipid programs (37).

##### Redox/KEAP1–NRF2 switch (stress sensor that biases toward keratinization)

Keratinocytes continually encounter endogenous ROS and electrophiles (mitochondrial leak, lipid-peroxidation adducts, UV by-products). These covalently modify KEAP1 cysteines, weakening CUL3-mediated NRF2 ubiquitylation; additionally, p62/SQSTM1 that accumulates during proteostasis stress can sequester KEAP1, stabilizing NRF2 (38, 39). Stabilized NRF2 accumulates in the nucleus with small Maf partners and binds ARE enhancers. Beyond classical antioxidant targets, epidermal NRF2 transcriptionally up-regulates keratinization/barrier genes (SPRRs, LCEs, IVL) and sulfur/thiol metabolism that supports disulfide-rich envelope assembly. *In vivo*, keratinocyte-restricted NRF2 activation drives hyperkeratosis and can rescue delayed barrier formation, illustrating that physiologic NRF2 activation biases differentiation/stratification (whereas chronic high activation overshoots) (40).

### Isolation of primary keratinocytes

Proper isolation epidermal KSCs that is fibroblast-free with minimal damage is a key step to ensure good supply of primary keratinocyte for cell culture. Obtaining skin biopsies from donors is the first step in acquiring and isolating primary keratinocytes. Various techniques and sources for skin biopsies are available and they are considered minimally invasive. Some of the most commonly used techniques are punch biopsy and shave biopsy (41). Alternatively, skin samples can be easily obtained during other medical interventions such as Cesarean patients, reconstructive abdominal plastic surgery, breast reduction and circumcision which is common to obtain neonatal cells. After harvesting the skin and soaking it in 70% ethanol for 1 min, samples should be immediately conserved in sterile pad soaked with saline solution or submerged is a saline solution. Commonly, Dulbecco's minimal essential medium (DMEM) or Minimum Essential Medium (MEM) supplemented with antibiotics and Fetal bovine serum (FBS) or fetal calf serum are used as a transport medium. Alternatively, HBSS or other media only supplemented with antibiotics can also be used. Tissue samples and transport media should be ideally kept at 4 C and samples can be stored for up to 24 h at 4 C in the transport medium before processing.

The processing of the skin sample starts by several washing steps typically with antibiotic supplemented media or PBS. Under sterile conditions, the skin samples are scraped off hypodermis, flattened and cleaned off all subcutaneous elements such as adipose tissue. This is followed by few more washing before moving to the Keratinocyte isolation step. Majority of protocols use enzymatic methods that helps in separating epidermis from dermis, and this can be either a one step or two step procedure (Table 1). The initially introduced one step method relies on trypsin alone for digestion of finely minced skin and withdrawing single-cell suspension every 30 min (42). Commonly, a trypsinization flask is used to help in decanting the cell suspension while preventing undigested tissue from being poured out. Further improvements on this method involves the use vortexing or magnetic stirring, with the later showing improved cell isolation yield an colony forming efficiency in recent studies (43). Type II collagenase is also another enzyme used in single step isolations where cell suspension is collected and plated after overnight incubation of skin samples (44). Additionally, a recently optimized enzymatic combination employing hyaluronidase and collagenase I has demonstrated superior performance in terms of cell yield and viability (45). This protocol involves the digestion of finely minced epidermal tissue using hyaluronidase and collagenase I, facilitating efficient release of keratinocytes while preserving stem-like characteristics. The method has shown promise particularly in adult skin processing, which remains more resistant to enzymatic dissociation.

**Table 1:** Comparison of keratinocyte isolation and primary culture methods.

Category	Isolation method & key reagents	Procedure	Comparative metrics	Ref.
One-Step Enzymatic	Trypsin Digestion (Trypsin typically 0.25%)	Minced skin digested in trypsin (37 °C) to obtain a single-cell suspension, collected at defined intervals.	Yield: ∼3–4 × 10⁶ cells/cm^2^ adult skin; Viability: ∼70%–85% (time-dependent); CFE: moderate, reduced by fibroblast co-isolation.	(42, 43)
	Collagenase II Digestion (Collagenase II)	Skin incubated overnight with collagenase II; cell suspension collected and plated.	Yield: increased vs static trypsin; Viability: comparable to standard trypsin; CFE: significantly increased colony numbers per 10⁴ cells.	(44, 45)
Two-Step Enzymatic	Trypsin-HBS+Trypsin (Trypsin-HBS, 0.05% trypsin-EDTA)	Dermo-epidermal separation with trypsin-HBS, followed by trypsin-EDTA.	Yield: moderate–high (protocol-dependent); Viability: variable, reduced with prolonged digestion; CFE: low–moderate.	(47, 48)
	Dispase+Trypsin (Dispase, 0.05% trypsin-EDTA)	Neutral protease dispase separates dermo-epidermal layers, followed by trypsin-EDTA digestion.	Yield: lower than whole-skin trypsin; Viability: higher than trypsin-only; CFE: increased due to reduced fibroblast contamination.	(48, 50)
	Thermolysin+Trypsin (Thermolysin, 0.05% trypsin-EDTA)	Thermolysin enables dermo-epidermal separation, followed by trypsin-EDTA.	Yield: ∼2–3 × 10⁶ cells/cm^2^; Viability: high; CFE: significantly higher than trypsin-only methods.	(49)
	Actinidin+Trypsin (Actinidin / kiwi protease)	Actinidin separates dermo-epidermal layers; combined with trypsin for single-cell suspension.	Yield: comparable to dispase-based methods; Viability: high (>85%); CFE: moderate–high.	(51)
	Dispase or Thermolysin+Accutase (recombinant enzyme mix)	Applied after epidermal separation, gentler enzymatic digestion.	Yield: high and reproducible in adult skin; Viability: high (>85%); CFE: preserved clonogenic capacity.	(52)
	Dispase or Thermolysin+TrypLE Select	Used post-separation in place of trypsin; animal-origin-free	Yield: comparable to trypsin; Viability: comparable to trypsin; CFE: largely preserved, slight reductions reported in some studies.	(52)
	Liberase Digestion+Trypsin (Liberase DH/TM, Trypsin–EDTA)	Liberase (TL/DH) to digest skin and release epidermis; subsequent Trypsin dissociation (optional)	Yield: slightly reduced initial recovery; Viability: comparable immediately, improved during culture; CFE: preserved over passages.	(55)
Alternative Methods	Skin Explant Culture (No enzymes used)	Keratinocytes migrate from plated skin fragments; subcultured to remove fibroblasts.	Yield: low initial cell numbers; Viability: high; CFE: high enrichment of clonogenic keratinocytes.	(50, 57)
	Selective Media Post-Digestion (Serum-free selective medium)	One-step digestion followed by media that enriches keratinocytes	Yield: moderate after initial digestion; Viability: high (>85%) following selective expansion; CFE: increased relative to non-selective culture	(56)

This table summarizes commonly used keratinocyte isolation and primary culture approaches, comparing their relative performance based on yield efficiency, colony-forming efficiency (CFE), scalability, and key technical limitations.

Improved two-step methods that are less fibroblast-contaminated with more cell yield have been later introduced (46). In the two-step method, dermo epidermal separation is achieved through a protease treatment before digestion with trypsin. While the two-step method separation can also be achieved with trypsin-HBS mixture, the usage of trypsin is associated with decrease in cell viability (47, 48). Hence, the use of neutral proteases such as dispase and thermolysin for dermo epidermal separation is favored (49). Actinidin-extracted from kiwi fruit- is another recently reported cysteine protease that was successfully implemented in the digestion and separation of dermis and epidermis (50, 51).

In addition to these established methods, several commercially available alternatives have been introduced for improved cell viability and xeno-free processing. TrypLE Select, a recombinant, animal-origin-free enzyme, serves as a gentler substitute for traditional trypsin. It is applied after dermo-epidermal separation and is reported to preserve cell surface epitopes better than conventional trypsin; however, it generally results in lower cell yields (52). Similarly, Accutase, another recombinant proteolytic enzyme mixture, has been employed for keratinocyte isolation due to its reduced proteolytic harshness and xeno-free composition (52). Although both TrypLE™ Select and Accutase® offer improved biocompatibility and reduced enzymatic damage relative to conventional trypsin, several studies have reported a modest reduction in keratinocyte yield when using these alternatives. Nonetheless, other investigations have demonstrated that TrypLE™ and Accutase® can achieve comparable cell yield and viability to those obtained with standard trypsin–EDTA digestion protocols (53, 54).

Liberase, a proprietary enzyme blend composed of highly purified collagenase isoforms and thermolysin, has also been used for primary keratinocyte isolation. It facilitates dermo-epidermal separation and partial tissue digestion with reduced variability and improved reproducibility compared to crude collagenase preparations. Depending on the specific formulation (e.g., Liberase DH or TM), it may be used alone or in combination with other proteolytic agents, and has demonstrated favorable outcomes in terms of keratinocyte viability, particularly in protocols involving adult human skin (55).

While the two-step methods are well-established and proved to work well for neonatal tissue, further improvement is still required for adult tissue isolations as it is more challenging. Furthermore, long and non-optimal digestion conditions in addition to enzyme batch differences can lead to low keratinocyte yield and viability. One proposed solution is the use of one step digestion isolation followed by a selective media that only enriches for keratinocytes, thus omitting the dermo-epidermal separation step and quickly processing the skin samples (56). Other techniques utilized for further enrichment of high-colony forming keratinocytes after isolation include density gradient centrifugation, gravity-assisted cell sorting, and cell sorting with specific marker antibodies (46). However, methods of isolation that don't rely on enzymatic digestion at all also been proposed and successfully implemented. In explant isolation method, keratinocytes migrate out of the plated skin explants and grow on the culture vessel which later on can be passaged out (57). The simple explant method takes advantage of the time lag between the migration of keratinocyte vs. fibroblast to isolate rapidly growing primary keratinocyte cells that are fibroblast-contamination free.

### Two-Dimensional (2D) monolayer and three-dimensional (3D) organotypic keratinocyte cultures

Currently, two general approaches are used for the 2D culture of keratinocytes *in vitro*: the first involves the use of a feeder layer of either murine 3T3 fibroblasts or human dermal fibroblast (HDF). The second, truly feeder-free strategy, combines serum-free, chemically defined media with a wide range of basement membrane–mimetic substrates and coatings, or even biomimetic hydrogels—to foster keratinocyte attachment, proliferation, and differentiation without the use of animal-derived feeder layers (Table 2) (58). Since 1975, the method described by Rheinwald and Green has been mainly employed to culture keratinocytes. This method is based on the co-culture of human keratinocytes that is in contact with irradiated, non-proliferating murine 3T3 fibroblast that acts as a feeder cell layer. The feeder cells support the growth of keratinocyte through a complex but still ill-defined mechanism. Even though various types of murine and human fibroblast have been reported, thee *in vitro*–stabilized murine 3T3-J2 cell line has the most established track record and is considered the gold standard in supporting keratinocyte culture. The feeder layer is prepared by exposing the 3T3 cells to high dose of gamma rays to render them non-proliferating. The keratinocytes then are seeded onto the monolayer of the irradiated 3T3 cells in a basal medium supplemented with fetal bovine serum (FBS) and other components. Additionally, a high dose of adenine is typically added to inhibit the proliferation of the contaminating fibroblast in the initial cultures (59). For further propagation, cells are passaged into new culture vessels prepared with 3T3 feeder monolayers. Rheinwald and Green's culture technique is well-established and has yielded positive results in clinic settings. However, the use of the feeder co-culture system brings some disadvantages and risks as it relies on animal derived cells and components such as FBS. This could expose the patients to toxins, zoonotic pathogens and immunogenic agents, hence limiting their use in treatments and other clinical applications. Therefore, efforts have been established to develop serum-free medium and to replace murine feeder cells with HDF for safe and effective culturing of keratinocytes.

**Table 2:** Overview of commonly used keratinocyte culture media systems.

Category	Method	Key components	Features	Intended use / application	Ref.
Feeder- Based	Murine 3T3-J2 Feeder	Irradiated murine 3T3-J2 fibroblasts; FBS; basal medium; high-dose adenine	CFE: High; Lifespan: High; Xeno-Risk: High; Diff. Potential: High	Regulatory Standard: Still the basis for Epicel® (the only FDA-HUD for massive burns). Research: Declining. being actively phased out in new clinical trials.	(58, 59)
	Human Dermal Fibroblast (HDF) Feeder	Irradiated or autologous HDFs; FBS; basal medium	CFE: High; Lifespan: High; Xeno-Risk: Moderate; Diff. Potential: High	Clinical: Moderate (mostly Europe). Used in clinics aiming for “Xeno-reduced” grafts. Research: Moderate.	(60)
	Collagen- Embedded HDF+Serum-Free Medium	Collagen-embedded dermal fibroblasts; chemically defined, serum-free medium	CFE: Moderate; Lifespan: Moderate; Xeno-Risk: Moderate; Diff. Potential: High	Clinical: Low. Research: High for 3D Organotypic models (Skin-on-a-chip).	(61)
	Plasma- Polymer Coated+Irradiated HDF	Plasma-polymer surface; irradiated dermal fibroblasts; serum-free medium	CFE: Moderate; Lifespan: High; Xeno-Risk: Low-Moderate; Diff. Potential: High	Research: Moderate (Surface engineering). Clinical: Low. Specialized for subconfluent cell delivery and transfer to wound beds; targets “xeno-free” expansion	(62)
	Recombinant-Protein Co-culture Medium	Recombinant human ECM proteins; serum-free co-culture medium	CFE: High; Lifespan: Moderate; Xeno-Risk: Low; Diff. Potential: High	Clinical: Emerging/High Growth. The target for modern “Phase I/II” bioengineered skin trials. Research: Rising.	(63)
	Surge SFM+Fibroblast Feeders	Surge SFM (chemically defined, serum-free); feeder fibroblasts	CFE: High; Lifespan: High; Xeno-Risk: Low; Diff. Potential: High	Clinical: Very Low. Research: Experimental; targets massive expansion without serum.	(64)
Feeder- Free / Serum- Free	Boyce & Ham MCDB-153	MCDB-153; low Ca^2+^ (0.1–0.3 mM); EGF, insulin, hydrocortisone, phosphoethanolamine, monoethanolamine; bovine pituitary extract (BPE)	CFE: Moderate; Lifespan: Moderate; Xeno-Risk: Moderate; Diff. Potential: Moderate	Status: Historical baseline. Parent of almost all modern commercial SFM development.	(65)
	Partially Defined Commercial Media	KGM (Lonza); DK-SFM, EpiLife® EDGS (Gibco) – all retain low levels of BPE or animal-derived supplements	CFE: Moderate; Lifespan: Moderate; Xeno-Risk: Moderate; Diff. Potential: Moderate	Research: Very High; the “workhorse” for 2D signaling and toxicity studies. Clinical: Low/Phasing out. Bovine Pituitary Extract (BPE) poses regulatory (TSE/BSE) hurdles; being replaced by xeno-free/fully defined alternatives in new ATMP pipelines.	(66, 67)
	CnT-07 (CELLnTEC)	Transferrin, trace elements, growth factors (fully defined); no BPE or serum	CFE: High; Lifespan: High; Xeno-Risk: Low; Diff. Potential: High	Clinical: High Growth. Frequently used in European ATMP production for corneal and skin repairs. Research: Very High.	(67, 68)
	EpiLife® + Supplement S7 (Gibco)	EpiLife® base medium; Supplement S7 (animal-origin-free growth factors)	CFE: High; Lifespan: High; Xeno-Risk: Low; Diff. Potential: High	Clinical: Moderate. Used in specialized “burn centers” for cell-spray therapies (e.g., ReCell alternatives). Research: High.	(69)
	ECM- Produced by Fibroblasts	Decellularized fibroblast ECM; DMEM/F12 or defined medium	CFE: Moderate-High; Lifespan: Moderate; Xeno-Risk: Low-Moderate; Diff. Potential: High	Research: Moderate/High. Vital for studying how the native dermal “niche” influences basement membrane assembly and KSC stemness. Clinical: Low/Experimental. Primarily used in “Self-Assembly” tissue engineering (e.g., LOEX)	(70)
	Recombinant Laminin Coatings	Recombinant human laminin isoforms (LN-421, LN-511); defined medium	CFE: High; Lifespan: High; Xeno-Risk: Low; Diff. Potential: High	Clinical: Emerging Gold Standard for iPSC-derived skin. Research: Dominant for stem-cell niche studies.	(71)
	Soft Hydrogel Substrates	Polyacrylamide or PDMS hydrogels tuned to ∼50 kPa; defined medium	CFE: High; Lifespan: Moderate; Xeno-Risk: Low; Diff. Potential: High	Clinical: None. Research: High for Mechanobiology (YAP/TAZ signaling).	(72)
	Ad-MSC Conditioned Medium	Adipose-MSC conditioned medium; defined basal medium	CFE: Moderate-High; Lifespan: Moderate; Xeno-Risk: Moderate; Diff. Potential: High	Clinical: Experimental. Research: Moderate; focused on paracrine signaling and non-enzymatic harvesting.	(73)
	“pop-Up” ePUK Method	Epilife medium (serum- and fatty acid–free); high-volume feeding	CFE: High; Lifespan: Moderate; Xeno-Risk: Low; Diff. Potential: High	Clinical: Experimental. Research: Moderate; focused on paracrine signaling and non-enzymatic harvesting.	(74)

This table summarizes representative keratinocyte culture media, highlighting their formulation characteristics, supplementation requirements, compatibility with feeder-free or xeno-free conditions, and intended applications.

As opposed to the murine 3T3 feeder system, an alternative HDF based feeder method has been developed to mitigate the use of animal derived cells. Similar to the 3T3 cells, post mitotic or irradiated HDF feeder layer is established before culturing the keratinocytes. Further efforts also reported the use of co-culturing system that utilizes non-irradiated autologous HDFs for keratinocyte expansion (60). This system is much safer and omits the need to test for infectious diseases, however, HDF feeder co-culture remains an undefined system due to the use of FBS which contains serum. Consequently, a research extension of this culture system further improved the conditions with the use of non-irradiated HDF layer and serum free media for keratinocyte expansion. Stark et al. demonstrated that primary human keratinocytes, when co-cultured atop collagen-embedded dermal fibroblasts in a serum-free, chemically defined medium, could form well-stratified, orthokeratinized epithelia expressing differentiation markers (keratins 1/10, involucrin, filaggrin) at par with traditional serum-containing systems (61). Building on this, Higham et al. (2003) enhanced keratinocyte attachment and expansion using plasma-polymer-coated surfaces combined with irradiated dermal fibroblasts, providing a chemically defined feeder layer alternative (62). Later, Mujaj et al. (2010) introduced a recombinant-protein-based, serum-free co-culture medium capable of supporting the parallel expansion of both keratinocytes and fibroblasts, further advancing defined culture systems without serum or animal-derived components (63). Most recently, Ghio et al. (2023) developed Surge SFM, a chemically defined, serum-free medium supplemented with fibroblast feeders, which maintains a proliferative K19^+^ epithelial stem cell population across passages, supports full epidermal stratification, and enables long-term graft persistence *in vivo* (64). Collectively, these advances reflect a progressive refinement of co-culture strategies toward clinically applicable, xeno-free skin regeneration platforms.

The development of clinically translatable keratinocyte culture systems began in earnest in 1983 with Boyce and Ham's landmark serum-free, feeder-free medium (MCDB 153), which replaced Rheinwald and Green's murine feeder- and serum-dependent method by leveraging low calcium (0.1–0.3 mM) to maintain proliferative undifferentiated states while suppressing fibroblasts (65). This formulation introduced defined supplements like epidermal growth factor (EGF), insulin, hydrocortisone, phosphoethanolamine, and monoethanolamine. However, it retained a critical limitation: optimal clonal growth required bovine pituitary extract (BPE), an undefined component prone to batch variability and safety risks 16. In subsequent decades, commercial media such as Lonza's KGM (based on MCDB-153), Gibco's DK-SFM (Defined Keratinocyte-SFM) and Gibco's EpiLife® EDGS reduced BPE dependency but did not eliminate it entirely; animal based components were used or BPE was retained in lower amounts alongside hormones and lipids in a “partially defined” environment (66, 67).

Significant progress occurred with media such as CELLnTEC's CnT-07, which were completely BPE-free, serum-free, and xeno-free, relying solely on chemically defined growth factors such as transferrin and trace elements. This supported strong keratinocyte expansion while preserving stem/progenitor phenotypes and yielding stratified epidermal constructs (67, 68). A further leap came with Gibco's EpiLife® base medium, combined with the animal-origin-free Supplement S7, creating a fully defined, xeno-free system that extends primary human keratinocyte lifespan and supports efficient expansion as confirmed in recent studies (67, 69).

Ongoing improvements focus on optimizing growth factor cocktails, fine-tuning calcium concentrations, and developing novel growth surfaces to enhance keratinocyte proliferation, clonogenicity, and downstream stratification. A recent study used fibroblast-produced extracellular matrix (ECM) for xenogeneic-free keratinocyte expansion (70). Proteomicly, The ECM closely resembled the core matrix composition of natural dermis. Indeed, the keratinocytes rapidly proliferated on these matrices, retaining their small sizes and expressing the early-stage markers. Further characterization revealed high colony forming efficiency compared to collagen I grown cells. Additionally, keratinocyte sheets grown on the novel matrix displayed more stable cell-cell junctions and demonstrated more robustness. Another effort has successfully developed a chemically defined, xeno-free, feeder free culture system using biologically relevant recombinant human laminins (LNs) as culturing surface (71). LN proteins are naturally occurring in the basement membrane; they contribute to the physical structure and also serve as ligands for cellular receptors and signaling. Several types of LNs have been identified in the basement membrane. In particular, LN-421 and LN-511 has shown to support the growth of keratinocytes *in vivo* as a replacement for a feeder lay-er. The laminin system showed comparable gene expression profile, colony-forming efficiency and differentiation capacity to the 3T3-coculture system. Because it is a serum and xeno free system, there are no batch-to-batch variances occurring when culturing the cells, which typically is the case in culture media containing FBS and bovine purity extract.

Keratinocyte culture on biomimetic substrates has emerged as a compelling alternative to traditional plastic surfaces. In a seminal 2021 study, researchers demonstrated that primary keratinocytes grown on soft hydrogels tuned to ∼50 kPa stiffness—mimicking the mechanical properties of skin—exhibited notably altered cell architecture, increased density, and enhanced nuclear biomechanics, alongside upregulated expression of mechanotransduction proteins like components of the LINC complex (72). When these mechanically conditioned cells were used to generate 3D epidermal models, the resulting tissue displayed improved stratification and organization compared to those derived from keratinocytes expanded on rigid plastic. This finding highlights how substrate stiffness alone—independent of biochemical cues—can profoundly influence keratinocyte behavior, suggesting that mechanically biomimetic culture surfaces may significantly enhance the physiological relevance and performance of engineered skin constructs.

Hassanzadeh et al. (2018) developed a novel feeder-free approach for culturing human keratinocytes using adipose-derived mesenchymal stem cell (Ad-MSC) conditioned medium, eliminating the need for animal-derived components or feeder layers (73). This method supported robust keratinocyte proliferation and the formation of multilayered epidermal sheets, with preserved expression of stem/progenitor markers (P63, K14, K19, and *α*6 integrin) alongside differentiation markers (K10, involucrin, and filaggrin). Marcelo et al. described the epithelial “pop-Up” keratinocyte (ePUK) method, which uses repeated high-volume feeding with serum- and fatty acid–free Epilife medium to harvest proliferative keratino-cytes in suspension from confluent cultures (74). The ePUK approach enriches for highly clonogenic, migratory cells in a truly feeder-free context, improving both expansion speed and graft take in experi-mental skin models.

### Limitations and translational bottlenecks

Despite significant advances in chemically defined keratinocyte culture systems, these platforms still face critical limitations that hinder their clinical translation. Current serum-free media often fail to consistently support proper epidermal stratification, producing thin or dysfunctional skin equivalents lacking the robust barrier function of native tissue (64, 75). Additionally, these systems remain prohibitively expensive due to their reliance on recombinant growth factors and specialized supplements. Different commercial media formulations (e.g., KGM-CD, EpiLife, DK-SFM) generate keratinocytes with distinct morphological and functional characteristics, including variations in proliferation rates, differentiation potential, and stem cell retention (76). The media-specific differences mean that cells cultured in one system frequently cannot be used interchangeably with those grown in another, complicating standardization across research and clinical applications.

Rapid expansion phases — commonly required to generate clinically relevant cell numbers — impose replication stress that increases the probability of both structural and sequence-level genomic lesions. Aneuploidy, the gain or loss of whole chromosomes, is a hallmark of genomic instability and is frequently observed in human pluripotent stem cells (hPSCs) during long-term culture (77). Although less documented in primary keratinocytes, the underlying mechanisms are highly relevant to any rapidly dividing epithelial population *in vitro*. Trisomy 12 is a particularly common whole-chromosome abnormality, often arising not from a single rare event but simultaneously in a high percentage of cells (∼2%) during critical transition passages. The lack of serum-derived protective factors and the reliance on simplified growth factor cocktails may exacerbate these mitotic errors (78). TP53 (p53) alterations merit particular attention in the context of *in vitro* keratinocyte expansion because TP53 mutant clones are present as frequent, small clones in normal human epidermis and because p53 dysfunction confers both replicative advantages and altered responses to DNA damage. Several lines of evidence indicate that (1) heterozygous or functionally inactivating TP53 mutations can permit clonal expansion of epithelial cells *in vivo*, (2) p53 loss or dysfunction in keratinocytes increases replication stress and can permit accumulation of structural genomic abnormalities, and (3) mutant p53 alleles may be positively selected under strong proliferative culture conditions (i.e., during rapid expansion), thereby creating a population-level enrichment of mutant clones that would be missed by crude phenotypic assays (79). These findings imply that culture processes that favour extreme or prolonged proliferative selection can inadvertently select for TP53-compromised cells or other mutations that confer growth advantage.

#### 3D culture

**Figure 2:**
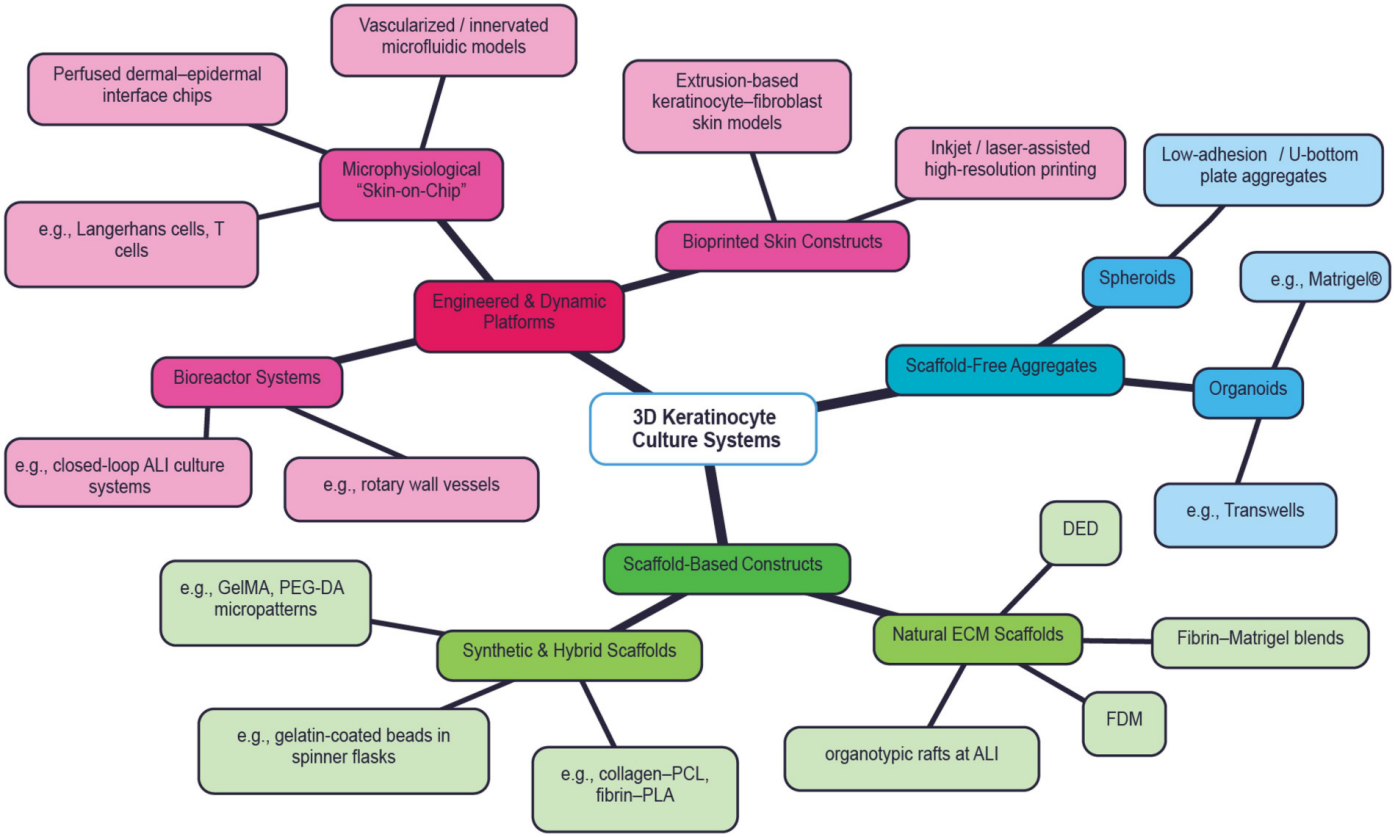
Overview of three-dimensional keratinocyte culture systems. Schematic representation of major 3D keratinocyte culture approaches, including scaffold-free aggregates, scaffold-based constructs, and engineered or dynamic platforms. Detailed features and representative examples are summarized in Table 3.

While the 2D culture of keratinocytes is well established and regarded useful in numerous research areas, the ability to grow them in a 3D culture systems is an important step forward towards understanding them in a more physiologically relevant environment. The 2D monolayer culture is limited as chemically defined systems are grown in low calcium concentration which prevents differentiation but impairs the formation of calcium-dependent adhesion structures (80). In contrast, Keratinocytes *in vivo* are connected on all sides by other cells with organized connected cytoskeletons that transduces mechanical signal between them. Hence, a 3D culture system of keratinocytes that can mimic the *in vivo* level of organization and connection is of importance (Figure 2).

**Table 3:** Classification and key features of 3D keratinocyte culture systems.

Major category	Subcategory	Platform/model type	Key features	Representative examples
Engineered & Dynamic Platforms	Microphysiological systems	Skin-on-chip models	Perfusion, controlled microenvironments, multi-cellular integration	Perfused dermal–epidermal interface chips; immune-competent skin-on-chip models
		Vascularized/innervated microfluidic models	Coupled epithelial–vascular–neural signaling	Vascularized and/or innervated microfluidic skin models
	Bioprinting platforms	Bioprinted skin constructs	Spatial control of keratinocyte–fibroblast architecture	Extrusion-based keratinocyte–fibroblast skin models
		High-resolution bioprinting	Precise cell placement and patterning	Inkjet- or laser-assisted bioprinting
	Bioreactor systems	Dynamic culture platforms	Controlled shear, oxygenation, scalability	Closed-loop ALI systems; rotary wall vessels
Scaffold-Free Aggregates	Spheroids	Cell aggregates	Self-assembly without exogenous scaffolds	Low-adhesion or U-bottom plate aggregates
	Organoids	3D epithelial structures	Self-organization and tissue patterning	Epidermal organoids (often Matrigel®-embedded); Transwell cultures
Scaffold-Based Constructs	Synthetic & hybrid scaffolds	Polymer-based hydrogels	Tunable mechanics and architecture	PEG-DA (inert); GelMA (adhesive); collagen–PCL; fibrin–PLA
		Microcarrier systems	High surface-area expansion	Gelatin- or collagen-coated microcarriers in spinner flasks
	Natural ECM scaffolds	Biologically derived matrices	Native biochemical and structural cues	Fibrin–Matrigel blends; decellularized dermis (DED)
		Organotypic cultures	Stratification and barrier formation	Organotypic rafts at air–liquid interface (ALI)
	Advanced fabrication	Additive scaffold manufacturing	Structured ECM deposition	Fused deposition modeling (FDM); directed energy deposition (DED)

This table provides a structured overview of current three-dimensional keratinocyte culture platforms, including engineered and dynamic systems, scaffold-free aggregates, and scaffold-based constructs. For each category, major subtypes, defining features, and representative examples.

Scaffold-free aggregates encompass both simple spheroids and more complex organoids, each preserving keratinocyte stem-like properties in 3D. Hanging-drop and low-adhesion/U-bottom plate spheroids formed under physiological calcium (1.2 mM) promote cell–cell contact around the entire aggregate without inducing terminal differentiation, maintaining P63^+^/K14^+^ stem markers despite the high calcium milieu (81, 82). Similarly, self-organizing “mini-skin” organoids generated in Matrigel®—which contain basal progenitors—and simpler keratinocyte aggregates seeded onto permeable membrane inserts (e.g., Transwells) can both be matured at an air–liquid interface (ALI), recapitulating stratified epidermal architecture while retaining proliferative capacity and multipotency (83).

Scaffold-based constructs range from natural ECM hydrogels to fully synthetic matrices and microcarrier systems. Collagen I hydrogels, whether as simple gels or as fibroblast-contracted organotypic rafts cultured at ALI, reliably produce stratified layers expressing K10, involucrin, filaggrin, and profilaggrin, faithfully modeling epidermal barrier formation (84). Fibrin–Matrigel blends and fibroblast-derived matrices (FDM) further support basement membrane assembly and epidermal differentiation (85), while decellularized dermis/DED scaffolds offer a native ECM backbone for keratinocyte repopulation and junctional complex formation (86). On the synthetic side, electrospun nanofiber mats (e.g., collagen–PCL, fibrin-coated PLA) guide keratinocyte migration into multi-layered, epidermis-like constructs, and tunable hydrogels such as GelMA or PEG-DA micropatterns allow precise control over stiffness and cell organization (87–89). Finally, gelatin-coated microcarrier beads expanded in spinner-flask yield high-density keratinocyte cultures that remain undifferentiated until subsequent ALI maturation (90).

Engineered and dynamic platforms integrate bioreactor control, 3D printing, and microfluidics to push epidermal modeling further. Suspension bioreactors—like rotary wall vessels—enable large-scale aggregate culture under low-shear conditions, while closed perfusion bioreactor systems, designed for culturing tissue-engineered skin at the air–liquid interface, support continuous medium flow via peristaltic pumps and have been shown to enhance viability of keratinocytes and fibroblasts on various scaffolds (91, 92). Similarly, airlift bioreactors with constant flow have been used to cultivate prevascularized, organotypic skin grafts on fibrin scaffolds, demonstrating that higher seeding densities of keratinocytes under perfusion improve epidermal layer formation and capillary-like network development (93). Bioprinted constructs, produced via extrusion-based layering of keratinocytes and fibroblasts or high-resolution inkjet/laser patterning, can be matured at the ALI to generate full-thickness skin equivalents with distinct basal, spinous, and granular layers expressing K14, K10, loricrin, and filaggrin (94). Microphysiological “skin-on-chip” devices integrate perfusable dermal–epidermal interfaces, vascular or neuronal co-cultures, and immune components (e.g., Langerhans cells, T cells) under microfluidic flow, producing barrier function and cellular crosstalk that closely mirror *in vivo* skin physiology (95).

### Assays to benchmark keratinocyte stemness and function

Methods of SC isolation are now well-stablished while the *in-vitro* culture systems of epidermal KSC are improving with new media formulations that are serum and Xeno free. Consequently, different assay has been developed to study the KSC and evaluate their capacity to self-renew, proliferate and differentiate. These assays are important in characterizing the SCs, and also assess their viability for grafting and other clinical applications.

The self-renewal and differentiation of epidermal KSCs are assayed using three different basic techniques. The first method examines the clonal growth ability of individual cells in culture through colony forming assays (CFA) or clonogenic assays (96). Cells are evaluated by culturing them at clonal density and then subcloned. Specifically, unlike undifferentiated cells that expresses SC markers differentiated cells can't form colonies (97). CFA of keratinocyte SC has led to identification of three types of clones, each demonstrating different proliferative potential. This offers another tool to monitor the comparative efficacy of different culture conditions in maintaining growth potential of SC by analyzing the clonal composition (98). The second method uses lineage tracing to genetically label a stem cell and trace it's progeny, which gives us information on how the cells behave in different conditions, the number of all progenies of the founder cell, their differentiation status and location. Using fluorescence labelling and other reporters, *in situ* monitoring and fate mapping of epidermal SC was possible in intact undamaged tissue (99, 100). Nowadays, large number of clones in complex tissues can be traced by using induced heritable DNA barcodes or naturally occurring somatic mutations that can be read using next generation sequencing (101).

The third method uses *in vitro* skin forming assay to assess the functionality of keratinocytes and their capacity to keratinize and form the epidermis. Simple epidermis reconstruction assays are achievable using cell culture inserts with porous membranes. After culturing the cell monolayer on the membrane, the inserts are lifted for air-liquid interface and the culture media is supplemented with extra calcium, thus facilitating the stratification of the cells and the formation of the epidermis layers.

Additional methods of assessment and characterization has also been described in literature. In wound-healing, migration of keratinocytes is essential for the complete reepithelization of the damaged site. Hence, a simple *in vitro* scratch-wound assay is utilized to assesses the migratory capacity of keratinocytes (102). Simply, cells are grown on a monolayer until 80% confluency. This is followed by removal of media growth factors and the mechanical disruption of cells by scratching the monolayer with a tool. By observing the cells under a microscope and periodically measuring the scratch gap, the migratory capacity of the cells can be evaluated.

*In vivo* methods for assessing Epithelialization, skin reconstitution and formation of hair follicles by keratinocytes has also been described (103–105). The tracheal grafting method involves inoculating rat tracheas with keratinocytes and fibroblasts from newborn mice then implanting back into the mice. The tracheas are then monitored for 2–4 weeks to study epithelialization and hair follicle reconstitution. The other method involves the injection of SCs into the dermo-epidermal junction of newborn mouse skin graft. The grafts are then placed and stitched on the back of athymic mice and monitored for epithelialization and hair growth. Diett et al. reported the use of a silicon chamber with a full thickness wound on the upper back of immunocompromised mice (106). The chamber is injected with dermal and epidermal SCs and the reconstituted tissue is assessed after two weeks. These methods allow researchers to investigate the dynamics of epithelialization and the formation of hair follicles *in vivo*, providing valuable insights into skin biology and potential applications in regenerative medicine.

### Keratinocyte-based therapeutics in wound healing

**Figure 3:**
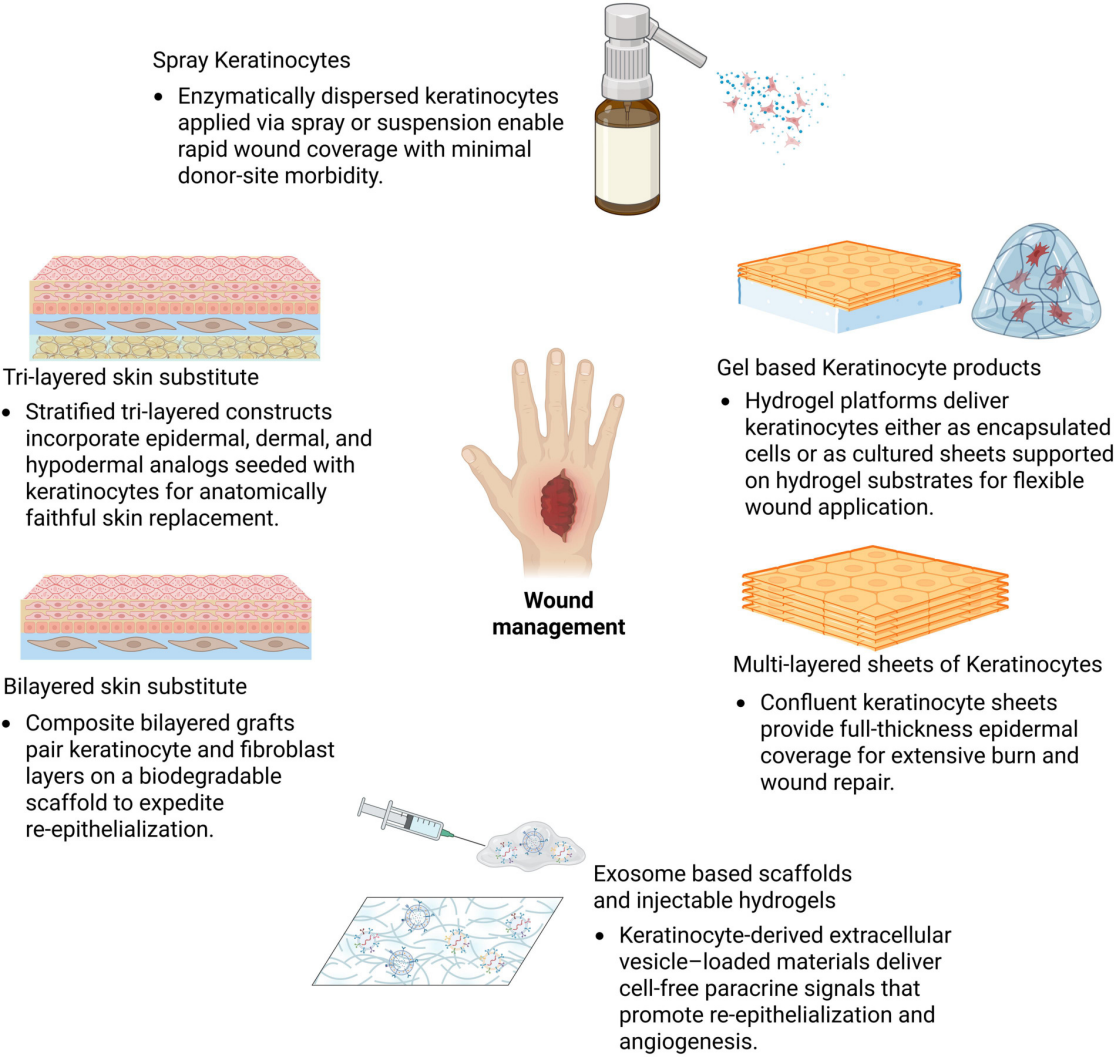
Keratinocyte-based approaches in wound management. Keratinocyte therapies range from cell-based grafts (autologous sheets and allogeneic composites) to cell suspensions and spray systems, hydrogel-based scaffolds, and acellular exosome-derived products. Each platform leverages keratinocyte biology to promote re-epithelialization, modulate inflammation, and restore barrier function in acute and chronic wounds. Together, these approaches demonstrate the evolution from traditional cultured epithelial autografts to next-generation, bioengineered, and cell-free regenerative skin therapies.

Since the introduction of cultured epithelial autografts (CEAs) in the early 1980s, keratinocyte-based therapies have become fundamental in modern burn care and wound management. Today, these products fall into four main categories—each designed to address specific clinical needs. In the following sections, we will briefly review representative products and the key advances within each category (Figure 3).

Solid Epidermal and Multilayered Keratinocyte-Based Sheets

Cultured keratinocyte sheets have long constituted the cornerstone of cell-based wound therapy. Autologous confluent sheets—such as Epicel®—are expanded from patient biopsies over 2–3 weeks, providing durable reconstitution of full-thickness epidermis in extensive burns (FDA-approved for ≥30% TBSA) with take rates around 75% (107). Off-the-shelf allogeneic composite sheets (Apligraf®, OrCel®, StrataGraft®) incorporate keratinocyte and fibroblast layers on biodegradable scaffolds, delivering metabolic and extracellular matrix cues that expedite re-epithelialization without permanent engraftment. More recently, fully biomimetic tri-layered skin substitutes—combining epidermal, dermal, and hypodermal analogs—have emerged. These constructs integrate keratinocyte-seeded hydrogels with fibroblast and adipocyte layers or basement membrane–mimetic films, aiming to replicate full skin architecture and improve outcomes in deep wounds (108). While still largely preclinical, these stratified platforms demonstrate promising re-epithelialization, vascularization, and appendage regeneration, heralding the next generation of durable, anatomically faithful skin replacements.

2. Keratinocyte Suspensions and Spray Systems

Keratinocyte suspensions and spray-based delivery systems represent a significant evolution in wound care, aiming to address limitations associated with sheet grafts by offering rapid treatment and reduced donor-site morbidity. Autologous approaches, such as the ReCell® system, harvest epidermal cells that are enzymatically dispersed and applied as a spray or suspension to partial-thickness wounds, donor sites, or ulcers (109). These preparations accelerate epithelialization and pigment restoration and significantly reduce donor-site burden, albeit with reduced structural support compared to sheet grafts. Adjunctive delivery methods, such as combining cell suspension with fibrin sealant (e.g., Vivostat®), have shown improved anchoring and take rates in challenging anatomical sites like the back (110). While autologous systems like ReCell® remain the mainstay, off-the-shelf allogeneic keratinocyte suspensions are under investigation to provide immediate biologic activity, though with less consistent engraftment.

3. Hydrogel-Based Cell Delivery Systems

Hydrogel-based platforms now represent a pivotal advancement in keratinocyte therapies, offering a bridge between rigid sheet grafts and fluid suspensions. Early studies utilized alginate hydrogels to encapsulate fibroblasts, with keratinocytes seeded atop to form bilayered constructs that maintain cell viability and aid full-thickness wound closure through scaffold degradation and extracellular matrix deposition (111). More recent innovations include polymerizable fibrin-based hydrogels embedding both keratinocytes and fibroblasts, enabling immediate *in situ* application without prolonged culture steps; these systems have demonstrated effective regeneration of skin architecture and accelerated healing in animal models (112). Additionally, tuning hydrogel stiffness has been shown to modulate keratinocyte proliferation and migration—cells expanded on softer hydrogels (10–20 kPa) retain superior wound-closure capacity compared to traditional plastic substrates (113).

4. Exosome/Secretome-Based Acellular Therapies

The latest wave in keratinocyte-centric approaches are exosome or secretome-derived products—cell-free systems delivering paracrine signals for repair. Keratinocyte-derived extracellular vesicles (EVs) promote re-epithelialization, angiogenesis, and modulation of inflammation. For instance, HaCaT-derived exosomal lncRNA MALAT1 has been shown to accelerate healing and promote M2 macrophage polarization in diabetic wounds. Similarly, mesenchymal stem cell and epidermal stem cell exosomes enhance collagen deposition, vascular ingrowth, and keratinocyte migration through PI3 K/Akt, Wnt/*β*-catenin, and macrophage reprogramming pathways (114). GelMA hydrogels loaded with keratinocyte-derived extracellular vesicles demonstrated accelerated angiogenesis and epithelial closure in diabetic mouse ulcer models via PI3 K/Akt pathway activation (115). These acellular formulations offer immediate therapeutic action, are stable, minimally immunogenic, and promising for diverse wound contexts without the complexities of living cell transplantation.

Clinical translation of keratinocyte-derived exosomal therapies is limited by the difficulty of achieving GMP-compliant, scalable manufacturing and by the intrinsic heterogeneity of extracellular vesicle preparations, which varies with cell source, culture conditions, and isolation methods. This variability complicates the establishment of robust identity, purity, stability, and batch-to-batch comparability criteria, while the absence of standardized, quantitative potency assays further challenges regulatory approval (116). Consequently, widespread adoption will require harmonized MISEV-guided characterization frameworks and validated GMP manufacturing pipelines tailored to EV-based therapeutics (116).

### Emerging applications beyond cutaneous wound care

**Figure 4:**
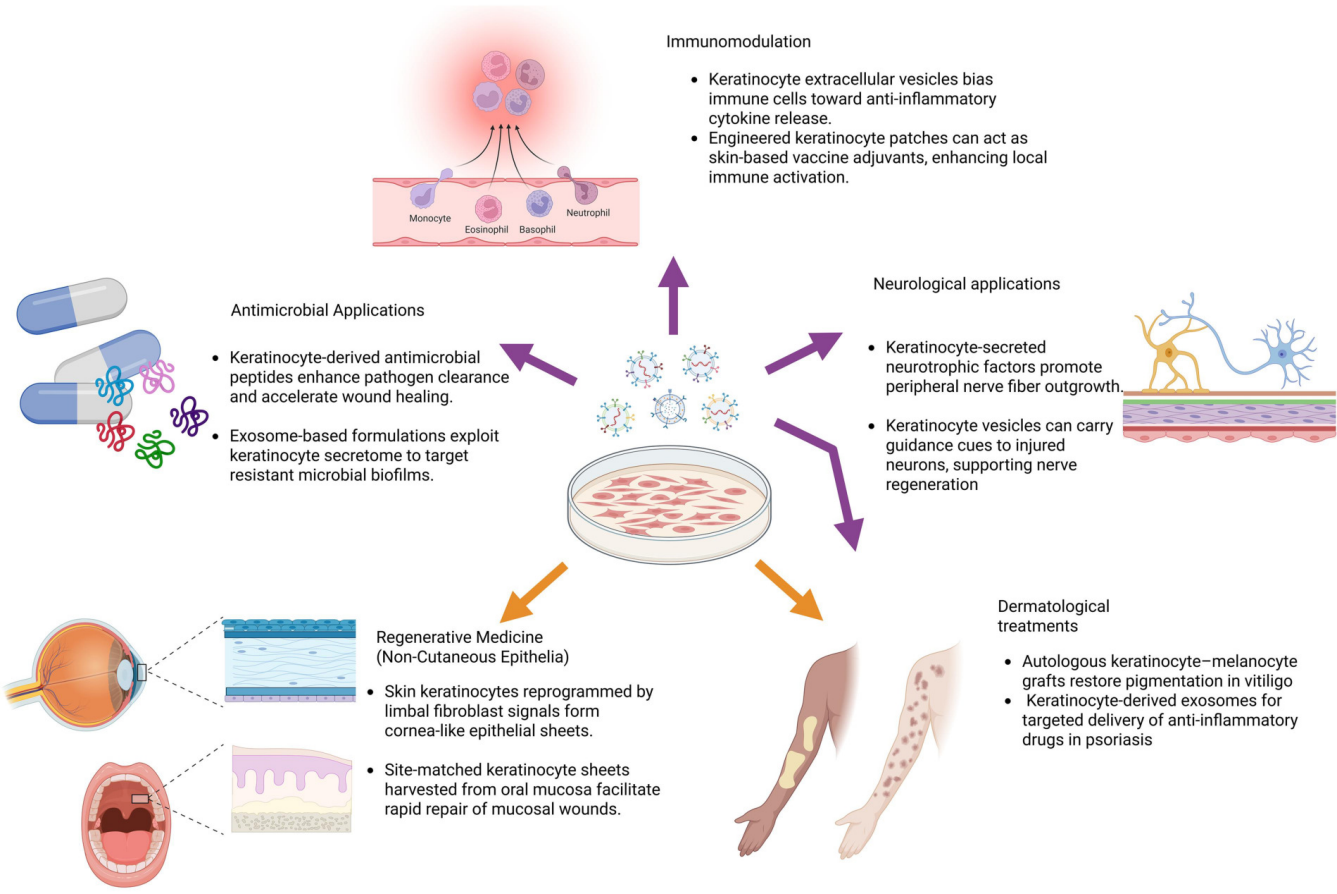
Emerging applications of keratinocytes beyond wound repair. Keratinocytes and their derivatives are being repurposed for diverse regenerative and therapeutic applications. These include dermatologic uses such as melanocyte–keratinocyte co-transplantation for vitiligo, immunomodulatory strategies employing engineered or exosome-delivered factors to treat inflammation and cancer, neuroregenerative approaches leveraging keratinocyte-derived trophic signals, and mucosal or ocular grafts for epithelial reconstruction. In addition, keratinocyte-derived antimicrobial peptides and exosomes are advancing as acellular biologics against infection. Collectively, these applications extend keratinocyte therapy from cutaneous regeneration to a broader, system-level regenerative medicine platform.

Since keratinocytes have historically been synonymous with grafts for burns and chronic wounds, it's easy to overlook their wider therapeutic potential. In reality, over the past decade researchers have begun harnessing both live keratinocyte transplants and their secreted products (exosomes, antimicrobial peptides, immunoregulatory vesicles, etc.) for applications as diverse as pigment restoration in vitiligo, modulation of autoimmune skin diseases, vaccine adjuvancy, peripheral nerve regeneration, ocular surface repair, and even targeted antimicrobial therapies. What follows is a concise overview of these cutting-edge, non-wound-care uses—each representing a novel frontier in keratinocyte biology and translational medicine (Figure 4).

Dermatology

Stable vitiligo, characterized by well-demarcated depigmented patches, has found effective treatment through autologous melanocyte–keratinocyte transplantation—a procedure that suspends patient-derived epidermal cells (both keratinocytes and melanocytes) and applies them to depigmented sites after skin preparation. Keratinocytes in this mix serve a crucial role, providing a nurturing microenvironment that supports melanocyte engraftment and melanin production—far beyond the simple replacement of lost cells (117). Over multiple clinical studies, approximately 36% of treated lesions achieve excellent repigmentation (≥ 75%–80%), with long-term data showing sustained results in over 50% of cases at 24 to 72 months post-treatment (118). A large retrospective analysis of 2,283 patients revealed excellent repigmentation in 58.8%–66% of cases, with higher success in segmental vitiligo, disease stability ≥ 6–12 months, and absence of Koebner (119). Overall, autologous melanocyte–keratinocyte transplantation has emerged as a robust, long-lasting, and well-tolerated alternative for stable vitiligo patients unresponsive to medical therapies. It achieves impressive aesthetic results through the natural synergy between keratinocytes and melanocytes and has been validated by both randomized controlled trials and extensive real-world outcomes extending beyond six years.

Researchers have begun repurposing keratinocyte-derived exosomes as a novel cell-free immunotherapy for inflammatory skin diseases like psoriasis. In a breakthrough preclinical study, ∼120 nm exosomes sourced from A-431 keratinocytes and loaded with the JAK inhibitor tofacitinib (“Exo-TFC”) displayed lower cytotoxicity and more potent suppression of psoriatic cytokine genes—TNF-*α*, IL-6, IL-23, and IL-15—compared to equivalent doses of free drug *in vitro* (120). When topically applied in an imiquimod-induced psoriasis mouse model, Exo-TFC induced superior lesion regression relative to free tofacitinib (120). This approach leverages inherently biocompatible keratinocyte vesicles, enabling efficient drug delivery, controlled release, and improved skin targeting through exosomal surface markers such as CD9. Additionally, keratinocyte exosomes have been shown to modulate neutrophil-mediated inflammation, although their effects can be pro- or anti-inflammatory depending on cellular context and stimuli (121). Although still in the preclinical stage, Exo-TFC exemplifies a paradigm shift, offering a promising, cell-free strategy for targeted immunomodulation in skin diseases—a novel frontier demonstrating how keratinocyte products can be engineered into next-generation dermatological treatments.

2. Immunomodulation

Secreted keratinocyte EVs (exosomes) tend to bias immunity toward non-specific responses. *In vitro*, keratinocyte exosomes internalized by dendritic cells induced IL-6 and IL-10 secretion (anti-inflammatory cytokines) but **not** TNF-α, and failed to drive antigen-specific T-cell proliferation (122). In other words, keratinocyte EVs resemble exosomes from *immature* rather than mature antigen-presenting cells, indicating inherent anti-inflammatory activity (122). Thus, keratinocyte-derived secretome or EVs are being explored (in preclinical models) as modulators of skin or systemic inflammation (for example, to dampen psoriasis or other autoimmune skin conditions). These strategies leverage keratinocyte products themselves, rather than whole grafts, to recalibrate immune responses.

Researchers have recently explored the innovative use of engineered keratinocytes as a skin-based vaccination platform. A compelling preclinical example involves keratinocytes genetically modified to overexpress the endoplasmic reticulum stress response factor XBP1, enabling them to create a locally pro-inflammatory skin environment that effectively enhances vaccination efficacy (123). In murine models, transient overexpression of XBP1 in epidermal keratinocytes triggered elevated production of cytokines and chemokines, recruited key immune cells—such as CD103^+^ dendritic cells, plasmacytoid dendritic cells, *γδ* T-cells, and innate lymphoid cells—and significantly amplified both antigen-specific cellular and humoral immune responses compared to antigen delivery alone. This translated into robust protection against Zika virus and induced therapeutic immunity in a melanoma model (124). The team further demonstrated that XBP1-modified keratinocytes generated a similarly immunogenic milieu in human skin explants, offering a strong foundation for translational potential.

Keratinocytes offer promising avenues for tumor immunomodulation by expressing key immune checkpoint molecules—particularly PD-L1 and the non-classical HLA-G1. Upon exposure to inflammatory signals like IFN-*γ* and TNF-*α*, these cells significantly upregulate PD-L1 and, to a lesser extent, HLA-G1, effectively inhibiting CD4^+^ T-cell proliferation through PD-1 engagement; PD-L1 blockade reverses this effect, underscoring its functional importance (125). Moreover, keratinocyte stem/progenitor populations (CD49f^high) exhibit heightened PD-L1 and HLA-G expression alongside secretion of TGF-*β* and IL-10—attributes resembling immunoprivileged cells—which suggests a role in creating a tolerogenic microenvironment (126). These findings suggest two translational strategies: (1) modifying keratinocyte expression of PD-L1 or HLA-G to locally modulate anti-tumor immunity—potentially improving immune surveillance in squamous cell carcinoma, and (2) exploiting keratinocyte-associated antigens, such as keratins 6, 14, and 17, which act as tumor-associated antigens and have been linked to better outcomes in non–small-cell lung cancer treated with checkpoint inhibitors. Together, these discoveries reveal keratinocytes as emerging tools in skin-targeted cancer immunotherapy, capable of both dampening and redirecting immune responses in the tumor milieu.

3. Neurological applications

Keratinocytes produce neurotrophic factors, such as nerve growth factor, together with defined epidermal paracrine mediators including brain-derived neurotrophic factor and glial cell line–derived neurotrophic factor family ligands, which regulate sensory neuron survival and neurite outgrowth (127). In culture, adult dorsal root ganglion, neurons extended axons more robustly when co-cultured with keratinocytes; keratinocyte-secreted factors significantly enhanced neurite outgrowth compared to neurons alone (128). Separately, keratinocyte-derived EVs have been shown to carry axon-guidance proteins and microRNAs; in diabetic neuropathy models, fluorescently labeled keratinocyte exosomes injected into skin were retrogradely transported to DRG neuron bodies (128). These preclinical findings (*in vitro* and mouse models) suggest that keratinocyte-conditioned media or exosomes could be developed to promote peripheral nerve regeneration after injury.

keratinocytes can also exacerbate nerve sensitization. In a rat model of nerve injury, implants of human keratinocytes at the injury site caused dramatic hyperexcitability of nearby neurons: the transplanted keratinocytes secreted excess NGF, leading to spontaneous firing and pain behaviors (129). This indicates a dual role: while certain keratinocyte factors encourage growth, they must be carefully controlled to avoid pathological pain. Understanding this keratinocyte–neuron crosstalk has opened potential for topical pain therapies (e.g., targeting keratinocyte NGF signaling) and warns that keratinocyte-based nerve grafts must be designed to prevent chronic pain (129).

4. Regenerative medicine (Non-Cutaneous Epithelia)

Keratinocytes are now being explored for cross-tissue regeneration, leveraging their shared ectodermal lineage with corneal epithelium. In a compelling proof-of-concept, human skin keratinocytes exposed to limbal fibroblast–conditioned medium underwent transdifferentiation, adopting corneal-specific markers CK3 and CK12, and forming stratified epithelial sheets closely resembling native corneal tissue *in vitro*; these engineered cells also exhibited reduced expression of angiogenic factors, which is critical for maintaining corneal clarity (130). This innovative strategy suggests that autologous skin keratinocytes—rather than skin or ocular grafts—could one day treat bilateral limbal stem cell deficiency by regenerating transparent corneal surfaces. Though still confined to laboratory studies, this application exemplifies a novel, cross-tissue use of keratinocyte grafting, shifting the focus from cutaneous repair to ocular tissue engineering and expanding the therapeutic horizon of keratinocyte-based regenerative medicine.

keratinocyte-based tissue engineering has also been successfully adapted for repair of oral and other non-cutaneous mucosal epithelia. Unlike skin-derived grafts, these approaches utilize keratinocytes directly harvested from mucosal tissues, such as buccal or tongue epithelium, preserving their innate compatibility with the target site. In one rodent model, autologous oral mucosal keratinocytes cultured as three-dimensional cell sheets integrated seamlessly into deep tongue wounds, promoting rapid re-epithelialization with reduced fibrosis and a mature p63^+^ basal layer reminiscent of normal oral mucosa (131). Similarly, application of these mucosal sheets to human intraoral surgical defects demonstrated faster healing, thicker epithelial coverage, and minimal scarring compared to conventional treatments (132). Notably, when transplanted to post-endoscopic submucosal dissection sites in the esophagus, these grafts virtually eliminated stricture formation and accelerated closure—highlighting their potential to repair mucosa in anatomically distinct regions. This “site-matched” strategy extends to the urinary tract, where oral keratinocytes seeded onto bladder-derived scaffolds reconstructed functional urethral mucosa in rabbits with durable epithelial architecture and reduced stricture risk (133). These findings underscore a paradigm shift: by sourcing keratinocytes from mucosal, rather than cutaneous, origins, researchers are expanding the regenerative reach of epithelial grafting into diverse non-skin tissues—leveraging cell-autonomous characteristics to rebuild mucosal barriers with fidelity and function.

5. Infectious and Antimicrobial Applications

Keratinocytes are a major source of innate antimicrobial peptides (AMPs)—notably human *β*-defensins and the cathelicidin LL-37—which not only directly kill bacteria and fungi but also facilitate wound repair by stimulating angiogenesis and keratinocyte migration (134). This endogenous antimicrobial function has been translated into clinical application: in a randomized, double-blind trial involving diabetic foot ulcers, topical synthetic LL-37 cream, administered twice weekly for four weeks, significantly accelerated granulation tissue formation and wound closure compared to placebo, while showing trends toward decreased levels of inflammatory cytokines (IL-1*α*, TNF-*α*) and bacterial load (135). This trial validates LL-37 as a keratinocyte-derived biologic therapy in real-world settings. Additionally, engineered strategies are emerging in the preclinical space, such as exosome-based systems designed to deliver AMPs or modulatory proteins—leveraging the keratinocyte secretome for antimicrobial and immunomodulatory effects aimed at tackling resistant infections and biofilms (136). These developments underscore a strategic move from cell grafts toward molecular, cell-free keratinocyte products that harness innate epidermal defences to modulate skin immunity and promote healing.

## Conclusion

Keratinocytes—long regarded as mere structural units of the epidermis—have emerged as central players in both fundamental regenerative biology and translational medicine. From their finely tuned homeostatic programs, governed by interlocking extrinsic and intrinsic “fate locks,” to the sophisticated *in vitro* systems that now recapitulate these dynamics, keratinocytes stand at the intersection of basic stem-cell science and clinical innovation. Advances in enzymatic and explant-based isolation methods, together with the maturation of xeno-free, chemically defined, and feeder-free culture systems, have transformed primary keratinocytes from delicate research tools into scalable, regulatory-compliant therapeutic platforms.

Beyond their established use in wound healing, keratinocyte-based constructs—ranging from autologous sheets to 3D bioengineered skin and hydrogel composites—demonstrate expanding clinical value, accelerating epithelial repair while restoring function and aesthetics. Parallel breakthroughs in organotypic and microphysiological “skin-on-chip” models now permit precise mechanistic study of epidermal biology, drug response, and disease modeling. Emerging applications continue to broaden the keratinocyte horizon: in dermatology for pigment restoration and immunomodulation, in oncology as localized immune modulators, in neurology for peripheral nerve repair, and in regenerative medicine for rebuilding mucosal and ocular epithelia.

Looking forward, integrating multi-omic profiling, biomimetic materials, and cell-free keratinocyte derivatives such as exosomes and secretomes will be key to translating keratinocyte science into next-generation therapies. The convergence of developmental signaling insights with bioengineering and precision-culture technologies promises a future in which keratinocyte-based interventions are not only curative for complex wounds but also foundational in diverse tissue-repair and immune-modulatory strategies.

## Author's Note

This paper represents the opinions of the author(s) and does not mean to represent the position or opinions of the American University of Sharjah.
